# The bacterial and fungal microbiomes of ectomycorrhizal roots from stone oaks and Yunnan pines in the subtropical forests of the Ailao Mountains of Yunnan

**DOI:** 10.3389/fmicb.2022.916337

**Published:** 2022-07-29

**Authors:** Qingchao Zeng, Xiaowu Man, Annie Lebreton, Yucheng Dai, Francis M. Martin

**Affiliations:** ^1^Beijing Advanced Innovation Center for Tree Breeding by Molecular Design, Beijing Forestry University, Beijing, China; ^2^School of Ecology and Nature Conservation, Institute of Microbiology, Beijing Forestry University, Beijing, China; ^3^College of Forestry, Beijing Forestry University, Beijing, China; ^4^Université de Lorraine, INRAE, UMR Interactions Arbres/Microorganismes, Centre INRAE, Grand Est-Nancy, Champenoux, France

**Keywords:** mycorrhizal symbioses, community structure, evergreen forest, season, host impact

## Abstract

Ectomycorrhizal (ECM) symbioses play an important role in tree biology and forest ecology. However, little is known on the composition of bacterial and fungal communities associated to ECM roots. In the present study, we surveyed the bacterial and fungal microbiome of ECM roots from stone oaks (*Lithocarpus* spp.) and Yunnan pines (*Pinus yunnanensis*) in the subtropical forests of the Ailao Mountains (Yunnan, China). The bacterial community was dominated by species pertaining to Rhizobiales and Acidobacteriales, whereas the fungal community was mainly composed of species belonging to the Russulales and Thelephorales. While the bacterial microbiome hosted by ECM roots from stone oaks and Yunnan pines was very similar, the mycobiome of these host trees was strikingly distinct. The microbial networks for bacterial and fungal communities showed a higher complexity in *Lithocarpus* ECM roots compared to *Pinus* ECM roots, but their modularity was higher in *Pinus* ECM roots. Seasonality also significantly influenced the fungal diversity and their co-occurrence network complexity. Our findings thus suggest that the community structure of fungi establishing and colonizing ECM roots can be influenced by the local soil/host tree environment and seasonality. These results expand our knowledge of the ECM root microbiome and its diversity in subtropical forest ecosystems.

## Introduction

Boreal, temperate and tropical forests are major terrestrial biomes and host a large part of the plant, animal and microbial diversity (Baldrian, 2017). Importantly, forests play a crucial role in global climate regulation (Pan et al., 2011; Pohjanmies et al., 2017; Mishra et al., 2020). Forest trees are tightly associated to soil microorganisms, including widespread beneficial mycorrhizal associations (van der Heijden et al., 2015). Ectomycorrhizal (ECM) fungi colonize up to 95% of tree root tips in temperate, boreal and subtropical forest ecosystems—biomes that make up much of the global terrestrial carbon sink. These root-colonizing fungi receive photosynthetically-fixed carbon from their host plants in exchange for plant growth-limiting soil nutrients, such as nitrogen and phosphorus, and they are key players in soil carbon sequestration. Apart from improving tree nutrition, symbiotic fungi also enhance plant resistance against abiotic and biotic stresses (van der Heijden et al., 2015; Nehls and Plassard, 2018; Dreischhoff et al., 2020). Together with other commensal and endophytic bacteria and fungi, ECM symbionts constitute the plant microbiome which significantly expands the genomic potential of the host as it serves functions that support host development, growth and nutrition (Berendsen et al., 2012; Turner et al., 2013; Berg et al., 2014; Beckers et al., 2017). Mycorrhizal roots represent an important niche for soil bacteria and fungi (Garbaye, 1994; Perotto and Bonfante, 1997; Bending et al., 2002; Frey Klett et al., 2007; Marupakula et al., 2016). However, the factors influencing the composition and structure of the microbial communities inhabiting ECM roots and their interactions are still poorly known. Given the importance of ECM fungi in tree biology and forest ecology, there is thus a need for a better understanding of the mechanisms driving the assembly of bacterial and fungal communities in ECM roots.

Izumi et al. (2007) identified that the most abundant cultivable bacteria found in *Pinus nigra* roots colonized by the fungal ECM fungi *Suillus variegatus* and *Tomentellopsis submollis* pertained to the *Pseudomonas*, *Burkholderia* and *Bacillus* genera. Further studies have shown that the zone adjacent to hyphal tips of arbuscular mycorrhizal fungi (AMF), the so-called hyphosphere, hosted a wide range of bacteria belonging to the Firmicutes, Actinobacteria, Bacteroidetes, Proteobacteria, Chloroflexi, Planctomycetes and Verrucomicrobia (Marupakula et al., 2016; Emmett et al., 2021). Bacterial populations were much higher in the rhizosphere compared to the hyphosphere and a striking shift in the bacterial composition between the hyphosphere and the rhizosphere has been reported (Andrade et al., 1997; Frey Klett et al., 2007). *Bacillus* and *Arthrobacter* were frequently found in the hyphosphere, while *Pseudomonas* dominated in the rhizosphere soil confirming the effect of mycorrhizal fungi on bacterial composition (Frey Klett et al., 2007; Gahan and Schmalenberger, 2015; Zhang et al., 2021). In *Betula pubescens*, it has also been shown that different ECM associations hosted distinctive bacterial and fungal microbiomes (Izumi and Finlay, 2011). Carbon compounds, such as trehalose, released by the fungal mycelium likely promote bacterial growth and the mycelial networks may facilitate bacterial co-migration in soil (Frey Klett et al., 2007; Nazir et al., 2010; Warmink et al., 2011). On the other hand, the direct access of ECM fungi to glucose released by root cells entails a competitive advantage over other soilborne microbes. Thus, the presence of ECM fungi can, in some circumstances, lead to suppressed growth and respiration by other soil microorganisms, such as saprotrophic fungi, thereby further increasing belowground carbon sequestration. In turn, bacteria have beneficial effects on symbiotic fungi; *Burkholderia* BS001 is able to protect its fungal host from the detrimental effect of antifungal agents (Nazir et al., 2014), whereas some mycorrhiza-associated bacteria produce compounds that are antagonistic to plant pathogens (Riedlinger et al., 2006; Frey Klett et al., 2007). *Pseudomonas* and *Bacillus* of the AMF hyphosphere mobilize soil inorganic phosphorus (Wang et al., 2016). Mycorrhizal helper bacteria play a role in diverse key functions, including germination of fungal propagules, promotion of mycelial growth, reduction of soil-mediated stresses and affect host recognition (Garbaye, 1994; Perotto and Bonfante, 1997; Bending et al., 2002; Frey Klett et al., 2007; Aspray et al., 2013; Marupakula et al., 2016; Zhang et al., 2021).

Biotic and abiotic factors strongly influence the composition of bacterial and fungal communities. Plant developmental stages shape the assembly patterns of the plant microbiome (Xiong et al., 2021a), including the community of ECM fungi. The composition of the microbiome inhabiting ECM roots is modulated by the host tree genotype and physiology, but also by rhizospheric microorganisms, soil properties and seasonality (Frey Klett et al., 2007; Izumi and Finlay, 2011; Bonito et al., 2014; Deveau, 2016). Analysis of the core bacterial microbiome of ECM roots suggested that it is not influenced by the species of ECM fungi, while roots play an important role in shaping its composition (Marupakula et al., 2016). However, the contribution and importance of tree species on the ECM microbiome composition remain poorly understood. A deeper knowledge of the mechanisms that govern the assemblage of microbial communities in ECM roots is needed to better understand and predict the greater ecosystem impacts of ECM associations. This includes determining how the host affects the microbiome composition, as well as how ECM fungi impact their associated microbes.

In a companion study, we surveyed by metabarcoding sequencing, the diversity and composition of soil bacteria and fungi in an old-growth forest, dominated by stone oaks (*Lithocarpus* spp.) and in a secondary Yunnan pine woodland in the subtropical Ailao Mountains in the Yunnan province, China (Zeng et al., 2022). Limited information exists on the alterations of soil microbiological characteristics in subtropical montane forests, while little is known of how the microbiome distribution pattern, life history and functional traits vary during forest replacement, e.g., old-growth *Lithocarpus* forest versus native pine woodlands. We therefore assessed the effect of forest replacement and other environmental factors, including soil horizons, soil physicochemical characteristics and seasonality (monsoon vs. dry seasons). We showed that tree composition and variation in soil properties were major drivers for both soil bacterial and fungal communities, with a significant influence from seasonality. ECM symbionts dominated the functional fungal guilds. Owing to the importance of the ECM fungal community, we aimed here to characterize the fungal and bacterial microbiomes of ECM roots from stone oaks and Yunnan pines. We have used culture-independent, high-throughput ribosomal DNA amplicon sequencing to investigate variation patterns in bacterial and fungal communities associated to ECM roots. We hypothesized that (1) tree species would be the primary factor shaping the ECM root microbiome due to host selection effects and (2) the microbial composition would differ with environmental factors, such as seasonality.

## Materials and methods

### Experimental design and sample collection

The sampling site was located in the Ailaoshan National Forest Ecosystem Research Station in Jingdong country, Yunnan Province (24°31′ N, 101°01′ E). The Ailaoshan lie between the southern and northern subtropical forest formations in a transition area. The most extensive forest ecosystem is a contiguous primary, old-growth broadleaf evergreen stone oaks (*Lithocarpus*) association which covers 75–80% of the region (Young et al., 1992). Two types of forests, dominated by *Lithocarpus* sp. and *Pinus yunnanensis*, were selected in this study. The stone oaks (*Lithocarpus*) old-growth forest, lies in a protected area of 5,100 ha of evergreen forest with a stand age of more than 300 years (Song et al., 2017). The upper canopy of the forest is 18 to 25 m high. Diameter at breast height (DBH) of the selected stone oaks ranged from 75 to 180 cm. The soils are loamy Alfisols (Chan et al., 2006; Wen et al., 2017). The nearby *Pinus* woodland replaced evergreen old-growth broadleaf stands about 55–65 years ago (Young and Wang, 1989). The pine woodland was located in an open, park-like area with a minimum of human interference. The DBH of the selected pine trees ranged from 65 to 95 cm. Although these two forest associations are located a dozens of km apart, they had few plant species in common. In this area, the annual mean temperature is 11.6°C and the mean annual precipitation is 1799 mm, 86% of which occurs during the wet season from May to October (Song et al., 2017). Sample collection in these two forests was performed in April and August 2020, corresponding to the end of the dry season and wet seasons, respectively.

Both stone oaks and Yunnan pines are known to form ECM associations. We collected ECM root samples in six plots, three located beneath stone oaks and three located beneath Yunnan pines. Individual plots were separated by ~100 m and the area of each plot was ~900 m^2^. In each plot, four individual adult trees, separated by 5 m to 15 m, were selected for ECM root collection. For each tree, two samples were collected at 1 m (north and south) from the base of the trunk. We sampled the ECM root tips and ECM root cluster found in a series of soil samples (20 × 20 cm, depth of 0–30 cm). In total, 96 samples were collected for microbial community DNA analysis (2 sites × 3 plots × 4 trees × 2 directions × 2 seasons). ECM root tips and clusters were separated from the main root system using forceps and a cutter, and any remaining soil particles attached to ECM roots or ECM root clusters (Supplementary Figure S1) were carefully removed using forceps and distilled water. ECM roots were distinguished based on external morphological characteristics (i.e., presence of a fungal mantle, forked pine roots, clustered roots). Between 10 to 100 ECM roots were harvested per replicate and snap frozen in dry ice until brought back to the Kunming Institute of Botany (KIB) where root samples were stored at −80°C prior to DNA extraction.

### DNA extraction, gene amplification and sequencing

Total DNA was extracted from ECM roots using a modified CTAB protocol (Maropola et al., 2015). In brief, 850 μl CTAB buffer (1 M Tris–HCl, 4 M NaCl, 0.5 M EDTA, 2% CTAB, 0.2% β-mercaptoethanol) was added to ECM roots and incubated at 65°C for 1 h, followed by the addition of 850 μl 24:1 (v/v) chloroform/isoamyl alcohol solution. The tubes were vortexed for 10 min and centrifuged (12,000 rpm, 10 min). The supernatant was collected in a new tube and 850 μl chloroform/isoamyl solution was added to the samples. Next, the tube was vortexed for 10 min and centrifuged (12,000 rpm, 10 min). Equal volumes of ice-cold isopropanol were added to the supernatants collected in a fresh tube and mixed by inverting, followed by incubation at-20°C for 24 h and centrifugation (12,000 rpm, 30 min). The supernatants were discarded and DNA pellets were washed twice with 800 μl 75% ethanol and eluted following centrifugation (12,000 rpm, 3 min). The DNA pellets were air-dried and then resuspended in 80 μl ddH_2_O before storing at-20°C. The concentration and quality of DNA were determined using the Nanodrop ONE spectrophotometer (Thermo Fisher Scientific, Waltham, MA, United States).

Bacterial and fungal communities were analyzed by high-throughput DNA metabarcoding sequencing. Bacterial communities were profiled by targeting a region of the 16S rRNA using the primer pairs 515F/806R. The composition of the fungal community was assessed by targeting the internal transcribed spacers (ITS). The ITS1 primers were used to amplify the samples collected during the dry and wet seasons, whereas the 16S and ITS2 primers were only used to amplify the samples collected during the wet season. We sequenced both the ITS1 and ITS2 regions using the primer pairs ITS1F/ITS2 and ITS86/ITS4, respectively (Caporaso et al., 2011; De Beeck et al., 2014). All PCR reactions were performed in 30 μl volume with 15 μl of Phusion^®^ high-Fidelity PCR Master Mix (New England Biolabs), 0.2 μM each of forward and reverse primers and 10 ng template DNA. PCR amplifications were performed using the following program: 1 min initial denaturation at 98°C, 30 cycles of 10 s at 98°C, 30 s at 50°C, and 30 s at 72°C, with a final 5 min elongation at 72°C. Libraries were generated using the Illumina TruSeq DNA PCR-Free Library Preparation Kit (Illumina, United States) following the manufacturer’s recommendations and index codes were added. The library quality was assessed using the Qubit 2.0 Fluorometer (Thermo Scientific) and on the Agilent Bioanalyzer 2,100 system. All samples were pooled in equimolar concentrations and subsequently sequenced on the Illumina NovaSeq platform with a paired-end protocol by Novogene Biotech Co., Ltd. (Beijing, China).

### Bioinformatic processing of the sequences

Amplicon data were analyzed using a combination of VSEARCH v2.13.3 and QIIME 1.9.1. softwares (Caporaso et al., 2010; Zgadzaj et al., 2016). Raw sequences were split based on their unique barcodes and the primer sequences, and low-quality sequences were trimmed off using an *in-house* script. Next, the paired-end sequences were merged using the USEARCH software v11.0.667 and the resultant sequences were quality-filtered. Singletons were removed using VSEARCH. The sequence reads were then clustered into operational taxonomic units (OTUs) at 97% similarity level using the UPARSE pipeline. Chimeric sequences that were identified using the reference-based methods were removed from the data. Representative sequences were classified using the BLAST algorithm with SILVA v.13.2 and UNITE v8.0 reference databases (Quast et al., 2013; Nilsson et al., 2019). Mitochondrial and chloroplast DNA sequences, as well as the OTUs with a total relative abundance of <0.00001 in all samples were discarded. The raw sequencing data have been submitted to the Sequence Read Archive under the accession number PRJNA820166 (16S and ITS2) and PRJNA782391 (ITS1).

### Statistical analysis

To assess alpha-diversity indices, a rarefaction step of sequence reads was performed to obtain the same amount of reads among samples, i.e., 29,715, 34,997 and 6,744 reads, corresponding to the lowest number of sequenced reads per sample, for 16S, ITS1 and ITS2 amplicons, respectively. The cumulative sum scaling (CSS) was used as a normalization method for beta-diversity analysis. A principal coordinate analysis (PCoA) was performed using the cmdscale function in the vegan packages. Bacterial and fungal alpha-diversity (observed OTUs and Shannon index) was calculated using QIIME. The observed significant differences were evaluated by one-way ANOVA (analysis of variance). Bacterial and fungal beta-diversity was estimated according to the Bray-Curtis distance between samples. Permutational multivariate ANOVA (PERMANOVA) statistical tests were performed using the R packages, vegan, with the adonis function having 999 or 1,000 permutations (Xiong et al., 2021b).

Venn diagrams were drawn using the OECloud tools (https://cloud.oebiotech.cn) to analyze the overlapping and unique OTUs between *Lithocarpus* and *Pinus* samples. Differential abundance between *Lithocarpus* and *Pinus* samples was assessed at phylum and class levels using the STAMP software and Welch’s tests followed by Benjamini-Hochberg FDR corrections (Parks et al., 2014). This analysis was also performed at OTUs level using the Edge’s generalized linear model (GLM) in the “edgeR” packages, with trimmed mean of M-values (TMM) normalization method and a threshold of significance set at *p* < 0.05 (Robinson et al., 2010). To identify the biomarkers of different tree species and sampling season, a linear discriminant analysis effect size (LEfSe) was employed (Wilcoxon value of *p* <0.05, logarithmic LDA (linear discriminant analysis) score > 2; http://huttenhower.sph.harvard.edu/galaxy; Segata et al., 2011). To elucidate the microbial interactions taking place in the two forest associations and at different sampling seasons, microbial association networks were created using the OTU tables. To reduce network complexity, OTUs present in all samples for bacterial and 40% or 60% samples for fungal communities were selected to construct the co-occurrence networks (Xiong et al., 2021b). Spearman’s correlation coefficient between two OTUs were considered statistically robust at *ρ* > 0.6 with a corresponding value of *p* of <0.01 (Barberan et al., 2014). The pairwise comparisons based on abundances were performed using the rcorr function in the “Hmisc” package and the value of *p* was adjusted using the Benjamini-Hochberg method (Zhu et al., 2019). Co-occurrence networks were obtained, with each node representing one OTU and each edge denoting a strong and significant connection. Network visualization and calculations of network topological properties (e.g., degree and modularity) were performed using the interactive Gephi platform (Xiong et al., 2021b). Cladograms for taxonomy were drawn using the R package Metacoder (Foster et al., 2017). All the statistical analyses for data were performed using the R software v3.6.0.

## Results

### Microbial diversity and networks in ECM roots

**Figure 1:**
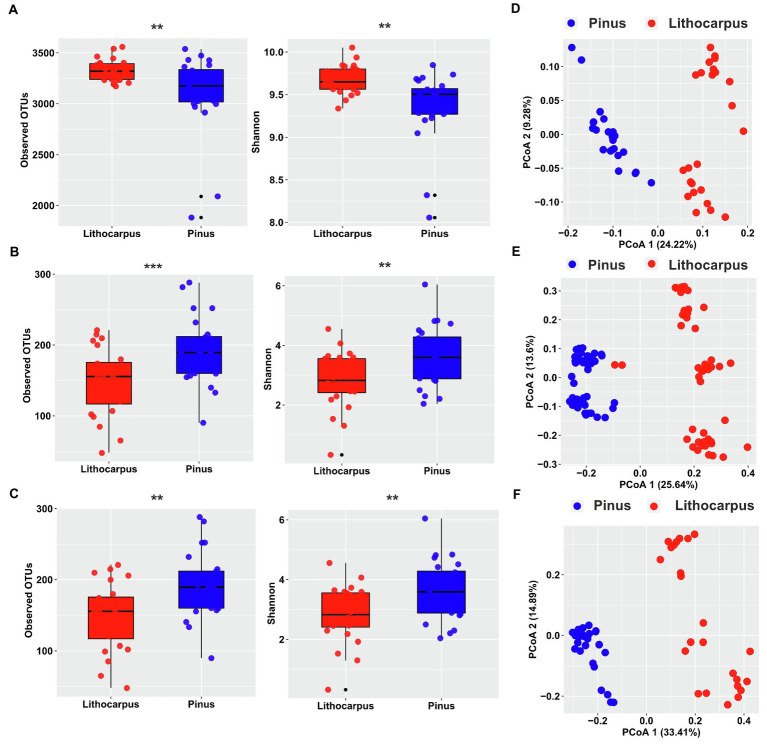
Diversity of bacterial and fungal communities associated to *Lithocarpus* and *Pinus* ECM roots. Alpha-diversity measurements are based on the observed OTUs and Shannon index for the bacterial **(A)** and fungal (**B**, ITS1; **C**, ITS2) microbiomes. Unconstrained PCoA for beta-diversity using Bray-Curtis distances in bacterial **(D)** and fungal (**E**, ITS1; **F**, ITS2) communities identified in *Lithocarpus* and *Pinus* ECM roots. Statistical data analyses were performed using one-way ANOVA (^**^p < 0.01, ^***^p < 0.001). For the alpha-and beta-diversity analyses based on ITS1 sequences we used samples collected during both the dry and wet seasons, while for the analyses based on ITS2 sequences we only used the samples collected during the wet season.

Bacterial and fungal communities associated to ECM roots of stone oaks and Yunnan pines were characterized by metabarcoding rDNA sequencing. In total, 4,791,454, 8,160,173 and 4,235,698 high-quality sequences were obtained and clustered into 6,081, 1,284 and 916 OTUs for 16S, ITS1 and ITS2, respectively. *Lithocarpus* ECM roots had a significantly higher bacterial alpha-diversity (observed OTUs and Shannon index) than *Pinus* ECM roots (*p* < 0.01, one-way ANOVA, Figure 1A). This bacterial alpha-diversity was positively correlated to the tree circumference (DBH; *p* < 0.001) for *Lithocarpus* ECM samples, whereas this was not the case for *Pinus* ECM roots (Supplementary Figure S2). Pine ECM roots displayed a significantly higher fungal alpha-diversity than *Lithocarpus* ECM roots (Figures 1B,C). No correlation between this alpha-diversity and tree circumference was observed (Supplementary Figure S2).

Both bacterial and fungal beta-diversities were mainly impacted by the host tree (*Lithocarpus* vs. *Pinus*; *R^2^* > 22%, *p* = 0.001). Seasonality also exerted a significant influence on the fungal community, but only accounted for a slight variation of the fungal OTU composition (9.79%; Figures 1D–F; Supplementary Table S1). The position of the soil sampling location relative to the tree trunk did not significantly influence the root microbiome (Supplementary Table S1).

**Figure 2:**
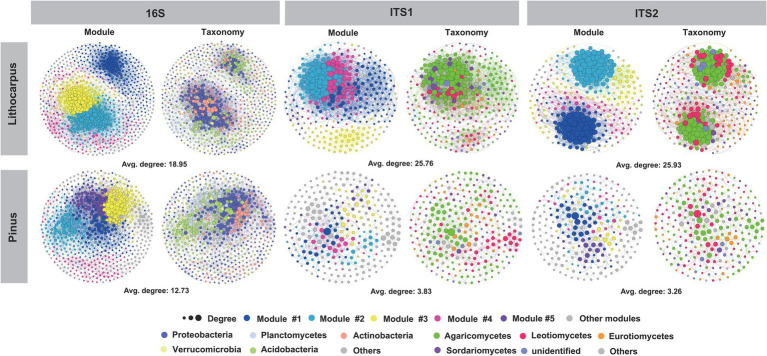
The co-occurrence networks of bacterial and fungal OTUs in *Lithocarpus* and *Pinus* ECM roots. The nodes in the network are colored based on phylum and class level or modularity class. The edge thickness is proportional to the weight of each correlation and node size is proportional to the degree of each OTUs. The co-occurrence network analysis based on ITS1 sequences have been carried out using samples collected during the wet season only.

To gain a deeper insight into the interactions among microorganisms, we performed a co-occurrence network analysis for each tree species. The microbial networks obtained for bacterial and fungal communities followed similar trends. Their complexity was higher in *Lithocarpus* ECM roots compared to *Pinus* ECM roots, but their modularity was higher in *Pinus* ECM roots (Figure 2; Table 1). We also detected more hub nodes in the networks associated with *Lithocarpus* ECM roots compared to *Pinus* ECM roots. Bacterial hub nodes pertained to the Actinobacteria and Proteobacteria phyla, whereas fungal hub nodes pertained to the Agaricomycetes class (Figure 2; Supplementary Table S2). The bacterial network associated with *Lithocarpus* ECM roots was enriched in OTUs pertaining to Planctomycetes (*p* < 0.05, fisher’s exact test, Supplementary Table S3). Fungal networks associated with *Lithocarpus* ECM roots were enriched in OTUs pertaining to Agaricomycetes (based on ITS1), whereas those associated with *Pinus* ECM roots were enriched in Eurotiomycetes and Archaeorhizomycetes OTUs (based on both ITS1 and ITS2).

**Table 1:** Topological properties of the co-occurrence networks of *Lithocarpus* and *Pinus* ECM roots.

Category	Average degree	Node	Edge	Modularity	Average clustering coefficient	Average path distance
16S						
*Lithocarpus*	18.946	810	7,673	0.503	0.443	5.46
*Pinus*	12.726	647	4,117	0.52	0.393	4.367
ITS1						
*Lithocarpus*	25.763	338	4,354	0.347	0.535	2.777
*Pinus*	3.833	276	529	0.791	0.552	5.763
ITS2						
*Lithocarpus*	25.931	319	4,136	0.534	0.649	4.017
*Pinus*	3.259	243	396	0.822	0.463	6.241

### Composition of bacterial and fungal communities of ECM roots

**Figure 3:**
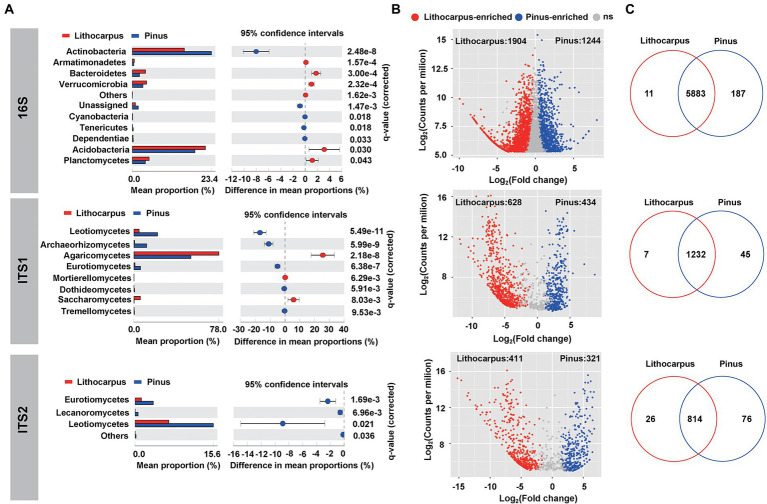
Tree species shape the microbial composition of ECM roots. **(A)** Differential abundance of bacterial and fungal OTUs in *Lithocarpus* and *Pinus* ECM roots. Welch’s tests followed by Benjamini-Hochberg FDR corrections were performed between *Lithocarpus* and *Pinus* ECM roots at phylum (bacterial OTUs) and class (fungal OTUs) levels. **(B)** The volcano plot shows the enriched OTUs in *Lithocarpus* and *Pinus* ECM roots. Each dot represents a single OTU. Each red dot represents an individual enriched OTUs in *Lithocarpus* ECM roots and each blue dot represents an individual enriched OTUs in *Pinus* ECM roots. The x-axis represents the fold-change in abundance and the y-axis represents the average OTUs abundance (in counts per million, CPM). **(C)** Venn diagrams showing the shared and specific bacterial and fungal OTUs among *Lithocarpus* and *Pinus* ECM roots. The ITS1 sequences used for this analysis were produced from samples harvested during both the dry and wet seasons.

The bacterial species identified in ECM roots belong to Proteobacteria (44.3%), Acidobacteria (20.3%), Actinobacteria (19.3%), Planctomycetes (4.5%) and Verrucomicrobia (3.7%). Rhizobiales (13.6%) and Acidobacteriales (13.2%) dominated this bacterial community leaving on the surface or inside ECM roots (Supplementary Figure S3). Pine ECM roots were enriched in Actinobacteria, Cyanobacteria, Tenericutes and Dependentiae with Actinobacteria being the most important biomarker taxa. *Lithocarpus* ECM roots were enriched in Acidobacteria, Bacteroidetes, Verrucomicrobia, Armatimonadetes and Planctomycetes with Acidobacteria being the most important biomarker taxa (Welch’s test, *p* < 0.05; Figure 3A; Supplementary Figure S4 A). Interestingly, *Pinus* ECM roots comprised a higher proportion of specific OTUs relative to *Lithocarpus*, while the latter had more significantly enriched OTUs (Figures 3B,C). OTUs annotated as Alphaproteobacteria, Actinobacteria, Thermoleophilia, Acidimicrobiia, Babeliae, Ktedonobacteria and Melainabacteria were more abundant in *Pinus* ECM roots, whereas Bacteroidia, Deltaproteobacteria, Verrucomicrobiae, Clostridia, Bacilli, Anaerolineae, Blastocatellia (subgroup 4) and Acidobacteria (subgroup 6) were more abundant in *Lithocarpus*-ECM roots (Fisher exact test, *p* < 0.05; Supplementary Table S4).

In the stone oak old-growth forest and the pine woodlands in Ailaoshan, ECM fungi, such as *Russula*, *Lactarius* and *Lactifluus* in the Russulaceae, were the most abundant OTUs detected in the soil fungal community, irrespective of the soil layers and season (Zeng et al., 2022). As shown by the present analyses, the fungal community of ECM roots was significantly different between the tree species and the sampling season had also a significant influence on this root-associated mycobiome (Figure 1; Supplementary Table S1). It was dominated by Agaricomycetes with a total relative abundance of 65 to 78% based on ITS1 or ITS2 sequencing, respectively (Supplementary Figures S5, S6). The most abundant OTUs associated to *Lithocarpus* roots belong to ECM taxa, such as *Russula*, *Tomentella* and *Laccaria*, whereas *Tomentella*, *Tylospora* and *Inocybe* were dominant in pine roots (Supplementary Figures S5, S6). Of note, a large proportion of OTUs comprised unidentified fungal taxa. In addition, Leotiomycetes, Archaeorhizomycetes, Eurotiomycetes, Dothideomycetes, and Tremellomycetes was significantly higher in stone oak ECM roots, whereas Agaricomycetes, Saccharomycetes and Mortierellomycetes were significantly lower in pine ECM roots (Welch’s test, *p* < 0.05; Figure 3A). Notably, we found substantial differences in OTU distributions according to the rDNA ITS region used for metabarcoding (ITS1 vs. ITS2; Supplementary Figures S7, S8). For example, the LEfSe analysis identified *Tomentella* in the Thelephoraceae (Supplementary Figure S4) as the main biomarker taxa for *Pinus* ECM roots, while the ITS1-based survey identified Leotiomycetes and *Archaeorhizomyces* taxa (Supplementary Figure S4). For *Lithocarpus* ECM roots, *Russula* was the major biomarker taxa based on both ITS1-and ITS2-sequencing.

Enriched ITS1-related OTUs affiliated to Russulaceae, Sebacinaceae, Boletaceae, Cortinariaceae, Hydnangiaceae, Tricholomataceae, Leotiaceae, Cephalothecaceae and Mortierellaceae were more abundant in *Lithocarpus*, while those annotated as Herpotrichiellaceae, Myxotrichaceae, Aspergillaceae, Inocybaceae, and Trichocomaceae were more abundant in *Pinus* (Fisher’s exact test, *p* < 0.05; Supplementary Table S5). On the other hand, enriched ITS2-related OTUs belonging to Russulaceae, Hyaloscyphaceae, Tricholomataceae, Cortinariaceae, Hydnangiaceae, Sebacinaceae and Gomphaceae were more abundant in *Lithocarpus*, while those corresponding to Atheliaceae, Myxotrichaceae, Inocybaceae, Trichocomaceae and Vibrisseaceae were more abundant in *Pinus* (Fisher’s exact test, *p* < 0.05; Supplementary Table S6). This discrepancy in OTU distribution related to the ITS sequences used for metabarcoding has been reported previously (De Beeck et al., 2014; Supplementary Figure S8) and thus, use of both ITS1 and ITS2 sequences are recommended for OTU surveys.

### Effect of seasonality on the fungal microbiome of ECM roots

**Figure 4:**
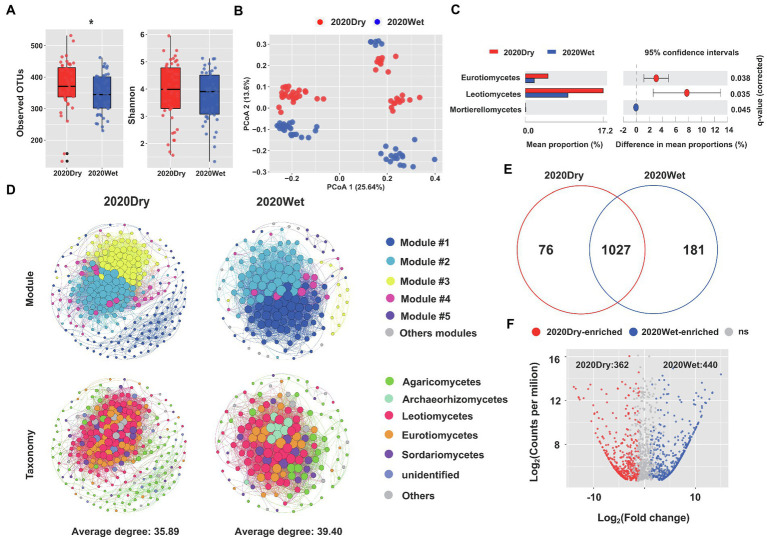
Seasonality influences the fungal microbiome of ECM roots. **(A)** Alpha-diversity measurements are based on the observed OTUs and Shannon index for the fungal OTUs. Statistical data analyses were performed using one-way ANOVA (^*^p < 0.05). **(B)** PCoA of beta-diversity using Bray-Curtis distances for fungal OTUs. **(C)** Differential abundances of fungal OTUs in ECM roots at the end of the dry season (2020Dry) and at the end of the wet season (2020Wet). Welch’s tests followed by Benjamini-Hochberg FDR corrections were performed for different sampling seasons. **(D)** Co-occurrence networks in fungal communities at the end of the dry season (2020Dry) and at the end of the wet season (2020Wet). **(E)** Venn diagrams showing the shared and specific fungal OTUs identified at the end of the dry season (2020Dry) and at the end of the wet season (2020Wet). **(F)** The Volcano plot displays the enriched OTUs for each sampling season. Each dot represents a single out, while red and blue dots represent an individual enriched OTUs identified at the end of the dry season (2020Dry) and at the end of the wet season (2020Wet), respectively.

The OTU richness and diversity of ECM roots sampled during the dry season were higher than those collected during the wet season. Notably, the observed OTU indexes for ECM roots harvested at the end of the dry season were significantly higher than those sampled at the end of the wet season (*p* < 0.05, one-way ANOVA; Figure 4A). Seasonality had a significant impact on beta-diversity (Supplementary Table S1). On our principal coordinate analysis (PCoA), the two main coordinates explained 39.24% of the variation, of which PC1 accounted for 25.64%, while PC2 for 13.6% of the total variation (Figure 4B).

The complexity of the microbiome networks taking place in ECM roots was higher during the wet season by comparison to the dry season, but the annotated nodes were not significantly different (Figure 4D; Supplementary Table S7). We found a higher abundance of Eurotiomycetes and Leotiomycetes and a lower abundance of Mortierellomycetes in dry season-samples compared to the wet season-samples (Welch’s test, *p* < 0.05; Figure 4C). The Leotiomycetes class was identified as the most significant marker of the dry season and the *Tomentella* genus was the most significant marker of the wet season (Supplementary Figure S9); 362 OTUs (e.g., Aspergillaceae, Trichocomaceae, Helotiaceae) and 440 OTUs (e.g., Thelephoraceae, Boletaceae, Cordycipitaceae, Amanitaceae and Gomphaceae) were significantly enriched in the ECM roots collected at the end of the dry season and wet seasons, respectively (Fisher’s exact test, *p* < 0.05; Figures 4E,F; Supplementary Table S8).

## Discussion

Forest trees are intimately associated with hundreds of microorganisms that contribute substantially to their biology. They are supra-organisms hosting a wide range of commensal, beneficial and detrimental bacteria and fungi. The tree with its associated microbiome—the collection of all microorganisms in a location—faces altered environmental conditions as a result of forest replacement and a rapidly changing climate. Characterizing the mechanisms shaping the tree microbiome is therefore required for a better understanding of tree fitness and adaptation to changing environments, and the ecology of forest ecosystems (Hacquard, 2016; Mishra et al., 2020; Hacquard et al., 2022). As tree roots are associated to hundreds of ECM fungi, there is a need to characterize the communities of bacteria and fungi inhabiting ECM roots (Garbaye, 1994; Perotto and Bonfante, 1997; Bending et al., 2002; Frey Klett et al., 2007; Marupakula et al., 2016). Several studies have characterized the bacterial communities associated with ECM roots (Izumi et al., 2007, 2008; Uroz et al., 2010; Vik et al., 2013; Nguyen and Bruns, 2015; Marupakula et al., 2016, 2017). On the other hand, little information is available on the fungal communities, i.e., the mycobiome, associated to ECM roots (Izumi and Finlay, 2011; Marjanović et al., 2020). In the present study, we surveyed both bacterial and fungal communities of roots from stone oaks and Yunnan pines, two dominant species of the subtropical forest associations found in the subtropical Ailaoshan. Our findings confirmed and extended previous studies in showing that the ECM microbiome is mainly shaped by the host tree. In addition, we found that seasonality had a significant effect on the fungal diversity and microbial network complexity of stone oak and pine ECMs.

### Distribution of bacterial and fungal OTUs in *Lithocarpus* and *Pinus* ECM roots

The diversity and composition of the bacterial microbiome of stone oak and Yunnan pine ECM roots were very similar with Actinobacteria, Acidobacteria and Proteobacteria dominating this community. Previous studies have also failed to demonstrate substantial differences between bacterial microbiomes associated with ECM roots (Burke et al., 2006; Izumi et al., 2007). In contrast, Izumi et al. (2007) have shown that the roots of *B. pubescens* colonized by various ECM fungi hosted distinct bacterial and ascomycetous communities. One explanation to explain the observed discrepancies between studies may be differential flow of carbohydrates and other nutrients from ECM fungi to the bacteria depending on the host tree species and age. The lack of any systematic variation in bacterial community structure related to the presence or absence of particular ECM fungal symbionts may also be related to the fact that temporal variation may confound systematic effects of the dominant mycorrhizal host fungi on bacterial community structure (Marupakula et al., 2016). The dominant orders found in *Lithocarpus* and *Pinus* ECM roots were Acidobacteriales, Rhizobiales, Betaproteobacteriales and Frankiales. Further studies will be required to elucidate the functional significance of these bacterial taxa but there is a broad correspondence between the groups found here and those identified in other studies of ECM roots. The presence of a core bacterial microbiome in ECM roots suggests that many of the bacterial genera were persistent in different ECM roots (i.e., from stone oak and pine), although there were changes in their relative abundance. This study highlights the diversity of bacteria associated with roots of major tree species growing in subtropical forests.

The fungal microbiome of stone oak and pine ECM roots was strikingly different at the OTU level, suggesting that the two tree species are recruiting a different set of ECM symbionts. As a consequence, this diversity impacts the cortege of non-symbiotic fungi inhabiting ECM roots. *Russula*, *Tomentella, Laccaria* and *Sebacina* taxa were the most abundant OTUs in *Lithocarpus* ECM roots and they are those forming the sampled ECM roots. OTUs belonging to commensal/saprotrophic fungi were also identified, including *Candida*, *Penicillium*, *Leotia* and *Oidiodendron* taxa. In *Pinus* ECM roots, the symbiotic *Tomentella* and *Tylospora* taxa were abundant, but a large proportion of OTUs belong to saprotrophic and endophytic taxa, such as *Archaeorhizomyces* and *Oidiodendron*. Tree phenology and thus leaf litter, but also metabolites released by roots, have a major impact on the rhizospheric microbial communities and recruitment of ECM symbionts (Frey Klett et al., 2007; Boberg et al., 2014), likely explaining the differences observed here in the distribution of ECM symbionts. In addition to root exudates, hyphal exudates, such as trehalose, released by the various ECM fungi played an important role in recruiting bacterial and fungal microbiomes (Zhang et al., 2021). We have shown in our companion study (Zeng et al., 2022) that the edaphic features, such as soil pH and nutrient content, were significantly different in the *Lithocarpus* old-growth forest by comparison to the *Pinus* woodland. These differences in edaphic features influenced the soil microbial communities (Zeng et al., 2022), but also the ECM microbial communities (present study) as shown in other forest ecosystems (Peay et al., 2013; Liu et al., 2014, 2015; He et al., 2017). Given that soil microbial communities feed the plant microbiome, both the reservoir of microorganisms in soil adjacent to roots and the soil edaphic parameters strongly influence the plant microbiome (Frey Klett et al., 2007; Bonito et al., 2014).

Our results showed that the alpha-diversity of the bacterial community from *Lithocarpus* ECM roots was significantly higher than that of *Pinus* ECM roots, while the trend was reversed for the fungal microbiome. Moreover, the microbial network occurring in *Lithocarpus* ECM roots was higher than that of *Pinus* ECM roots with a significantly different OTU composition (Figures 1, 2). A positive relationship between the alpha-diversity and tree circumference of *Lithocarpus* for the bacterial community was observed (Supplementary Figure S2). Of note, it has been reported that the ECM alpha diversity is not strongly linked to tree growth, while variations in the beta diversity of ECM community is the strongest predictor of tree growth rate across Europe (Anthony et al., 2022).

### Seasonality influences the ECM root mycobiome

The Ailaoshan resides in the center of the largest subtropical land area in the world (Young and Wang, 1989). This region is a major climatic border between China southwestern and southeastern monsoon systems, and the northern Himalaya Plateau. Influenced by the southwest and southeast monsoons, the climate of the Ailaoshan alternates between wet and dry conditions. Mean annual precipitation is 1799 mm, 86% of which occurs during the monsoon rainy season from May to October (Song et al., 2017). Therefore, seasonal variability in soil water content is substantial (Wu et al., 2014) and influences the soil microbial communities (Zeng et al., 2022). As soil water content affects the soil mycobiome composition (Zeng et al., 2022) and competition between ECM and saprotrophic fungi (Baldrian, 2017), we surveyed the ECM root mycobiome at the end of the wet or dry seasons. The alpha-diversity of the fungal community of ECM roots at the end of the dry season was higher than at the end of the wet season. Furthermore, samples collected during the dry season showed a higher abundance of saprotrophic Eurotiomycetes and Leotiomycetes, while ECM roots collected during the wet season were enriched in saprotrophic Mortierellomycetes. The observed seasonal changes in the ECM root mycobiome may be linked to variation in root metabolic activities. Frequent soil droughts taking place during the dry season may also favor ECM root senescence and decay, providing favorable conditions for the establishment of saprotrophic fungi.

### Fungal identification biases

As reported in previous studies, the recovered taxonomic richness of the fungal community differs among selected barcode-primer pair combinations for amplifying the rDNA ITS (De Beeck et al., 2014; Tedersoo et al., 2015; Mbareche et al., 2020). ITS1 and ITS2 sequencing provided consistent results in ranking taxonomic richness and recovering the importance of tree species in driving fungal community composition in stone oak and pine ECM roots, except for the Archaeorhizomycetes and Tremellomycetes which were mainly detected by ITS1 sequencing and Lecanoromycetes was more abundant in ITS2 sequencing (Supplementary Figures S7, S8).

## Conclusion

In conclusion, the present study provides new information about the identity and diversity of different bacterial and fungal microbiomes associated with different types of ECM associations from two major native tree species of a subtropical mountain forests. As reported previously, our results confirmed the major influence of the tree species on the composition of the ECM mycobiome. On the other hand, the bacterial communities associated with ECM roots were less influenced by the changes in the host tree and associated ECM fungal communities. This work paves the way for more detailed studies of the function expressed by the communities of ECM fungi and their associated communities of bacteria and fungi. A better understanding of the interactions between bacteria and fungi in the mycorrhizosphere of stone oaks and Yunnan pines will be critical to understand the soil–plant interface in the threatened subtropical forests.

## Data availability statement

The datasets presented in this study can be found in online repositories. The names of the repository/repositories and accession number(s) can be found in the article/Supplementary Material.

## Author contributions

QZ and XM collected the samples and extracted DNA. QZ analyzed the data with help from AL and XM. FM and YD coordinated the project and designed the experimental plan. QZ and FM wrote the manuscript with input from AL. All authors contributed to the article and approved the submitted version.

## Funding

This work was supported by the Beijing Advanced Innovation Center for Tree Breeding by Molecular Design and the Laboratory of Excellence ARBRE (grant number ANR-11-LABX-0002-01). We also acknowledge grants from the China Postdoctoral Science Foundation (2019M660508 to QZ), the National Natural Science Foundation of China (grant number U1802231 to YD), and the Second Tibetan Plateau Scientific Expedition and Research Program (STEP, No. 2019QZKK0503 to YD).

## Conflict of interest

The authors declare that the research was conducted in the absence of any commercial or financial relationships that could be construed as a potential conflict of interest.

## Publisher’s note

All claims expressed in this article are solely those of the authors and do not necessarily represent those of their affiliated organizations, or those of the publisher, the editors and the reviewers. Any product that may be evaluated in this article, or claim that may be made by its manufacturer, is not guaranteed or endorsed by the publisher.

## Supplementary material

SUPPLEMENTARY FIGURE S1 Different morphotypes of ECM roots sampled beneath *Lithocarpus* and *Pinus* trees in the Ailaoshan old-growth forest and pine woodland and the protocol used for the DNA metabarcoding survey.

SUPPLEMENTARY FIGURE S2 Linear regression showing the relationship between the tree circumference (BDH) and alpha-diversity of bacterial and fungal OTUs (observed OTUs and Shannon index).

SUPPLEMENTARY FIGURE S3 Relative abundance of the most abundant bacterial OTUs in *Lithocarpus* and *Pinus* ECM roots at phylum and order levels. The average relative abundance phyla or class with less than 0.05% across all samples are grouped into “Others”. **(A)** At phylum level, **(B)** At order level.

SUPPLEMENTARY FIGURE S4 The biomarker taxa of bacterial and fungal communities varied among *Lithocarpus* and *Pinus* ECM roots. LEfSe identified the biomarker taxa associated with *Lithocarpus* and *Pinus* ECM roots, respectively. Only the top 10 most specific biomarker taxa are displayed. **(A)** 16S rDNA sequencing, **(B)** ITS1 sequencing, **(C)** ITS2 sequencing.

SUPPLEMENTARY FIGURE S5 Relative abundance of the most abundant fungal OTUs in *Lithocarpus* and *Pinus* ECM roots at class, order and family levels based on ITS1 sequencing. The average relative abundance order or family with less than 0.05% across all samples are grouped into “Others”. **(A)** At class level, **(B)** At family level, **(C)** At genus level.

SUPPLEMENTARY FIGURE S6 Relative abundance of the most abundant fungal OTUs in *Lithocarpus* and *Pinus* ECM roots at class, order and family levels based on ITS2 sequencing. The average relative abundance order or family with less than 0.05% across all samples are grouped into “Others”. **(A)** At class level, **(B)** At family level, **(C)** At genus level.

SUPPLEMENTARY FIGURE S7 The choice of ITS region impacts the identification of fungal OTUs of ECM roots. **(A)** Relative abundances of the enriched fungal taxa using ITS1 and ITS2 primers at the class level. The average relative abundant classes less than 0.05% across all samples are grouped into “Others”. **(B)** Differential abundances of fungal OTUs identified by using ITS1 or ITS2 sequences. Welch’s tests followed by Benjamini-Hochberg FDR corrections were performed among different ITS. For these analyses, we used samples collected during the wet seasons.

SUPPLEMENTARY FIGURE S8 Taxonomic distribution of the most abundant fungal OTUs identified using either ITS1 or ITS2 sequences. Blue and red circles represent the microbial taxonomic ranks with are found as significantly more abundant based on ITS1 or ITS2 sequences, respectively. The color of nodes represents the value of the Log_2_ ratio of ITS1 and ITS2 with OTU count (Wilcoxon Rank Sum test, *P*<0.05). Only the OTUs with an average relative abundance >0.1% are displayed. For these analyses, we used samples collected during the wet seasons.

SUPPLEMENTARY FIGURE S9 The biomarker taxa identified in ECM roots at the end of the dry season (2020Dry) and at the end of the wet season (2020Wet) based on ITS1 sequencing. Only the top 10 most specific biomarker taxa are displayed.

SUPPLEMENTARY TABLE S1 Effects of multiple biotic and abiotic factors on the root microbiome composition of *Lithocarpus* and *Pinus* ECM roots.

SUPPLEMENTARY TABLE S2 Comparison of nodes assignments between *Lithocarpus* and *Pinus* ECM roots.

SUPPLEMENTARY TABLE S3 The hubs of microbial co-occurrence networks of *Lithocarpus* and *Pinus* ECM roots.

SUPPLEMENTARY TABLE S4 Comparison of enriched bacterial OTUs between *Lithocarpus* and *Pinus* ECM roots based on 16S sequencing.

SUPPLEMENTARY TABLE S5 Comparison of enriched fungal OTUs between *Lithocarpus* and *Pinus* ECM roots based on ITS1 sequencing.

SUPPLEMENTARY TABLE S6 Comparison of enriched fungal OTUs between *Lithocarpus* and *Pinus* ECM roots based on ITS2 sequencing.

SUPPLEMENTARY TABLE S7 Comparison of node assignments between ECM roots sampled at the end of the dry season (2020Dry) or at the end of the wet season (2020Wet) based on ITS2 sequencing.

SUPPLEMENTARY TABLE S8 Comparison of node assignments between ECM roots sampled at the end of the dry season (2020Dry) or at the end of the wet season (2020Wet) based on ITS2 sequencing.
