# Supplementary figures and images for: The bacterial and fungal microbiomes of ectomycorrhizal roots from stone oaks and Yunnan pines in the subtropical forests of the Ailao Mountains of Yunnan
Source: Front Microbiol. 2022 Jul 29;13:916337. doi: 10.3389/fmicb.2022.916337 (PMC9372452; doi:10.3389/fmicb.2022.916337)

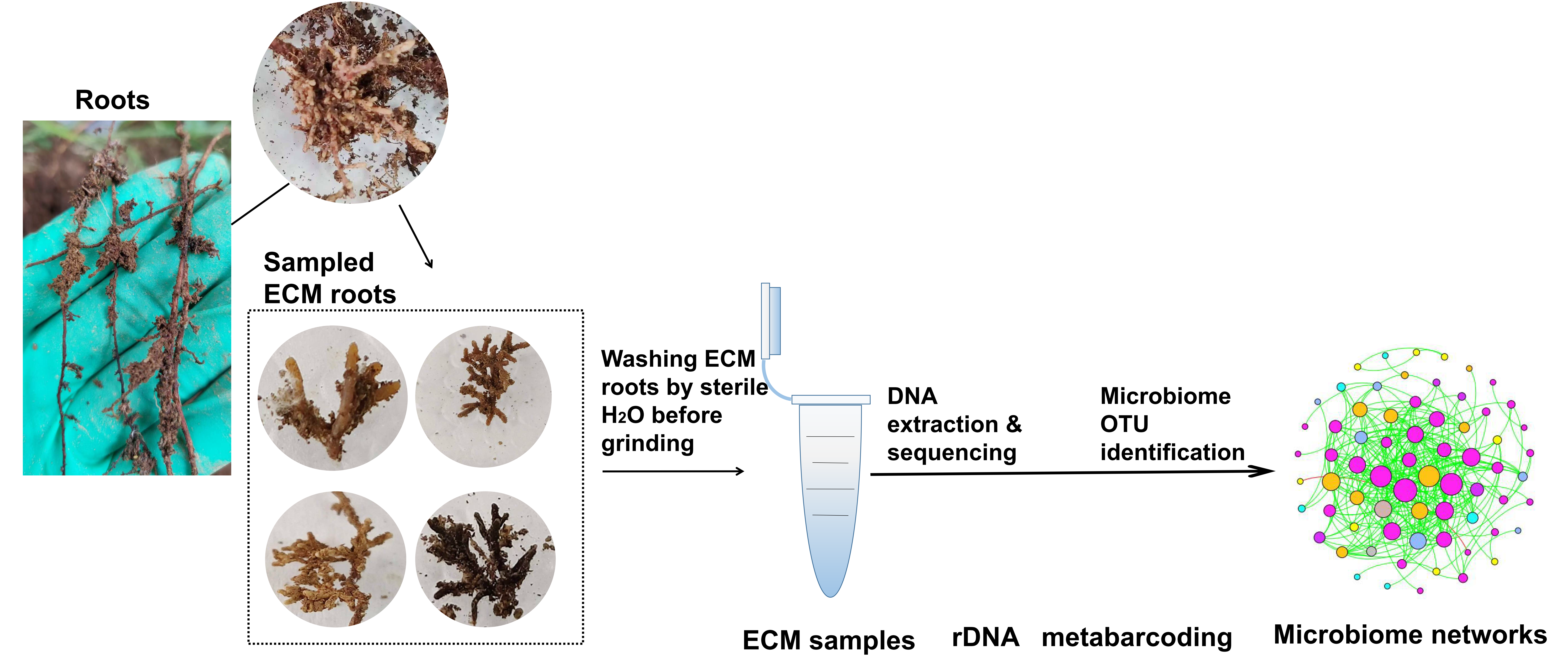

Supplement: SUPPLEMENTARY FIGURE S1 — Different morphotypes of ECM roots sampled beneath Lithocarpus and Pinus trees in the Ailaoshan old-growth forest and pine woodland and the protocol used for the DNA metabarcoding survey. [file Image_1.TIF]

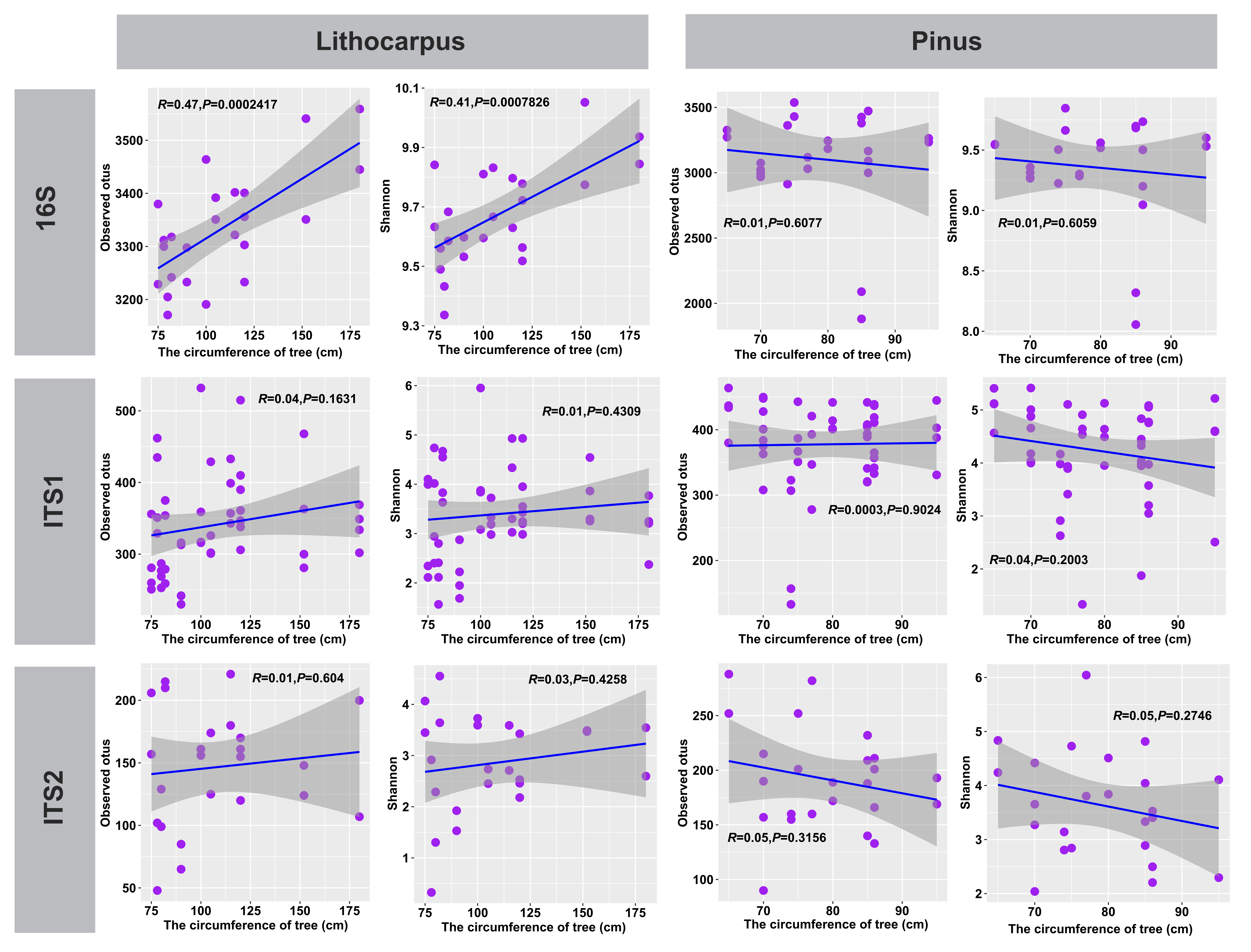

Supplement: SUPPLEMENTARY FIGURE S2 — Linear regression showing the relationship between the tree circumference (BDH) and alpha-diversity of bacterial and fungal OTUs (observed OTUs and Shannon index). [file Image_2.TIF]

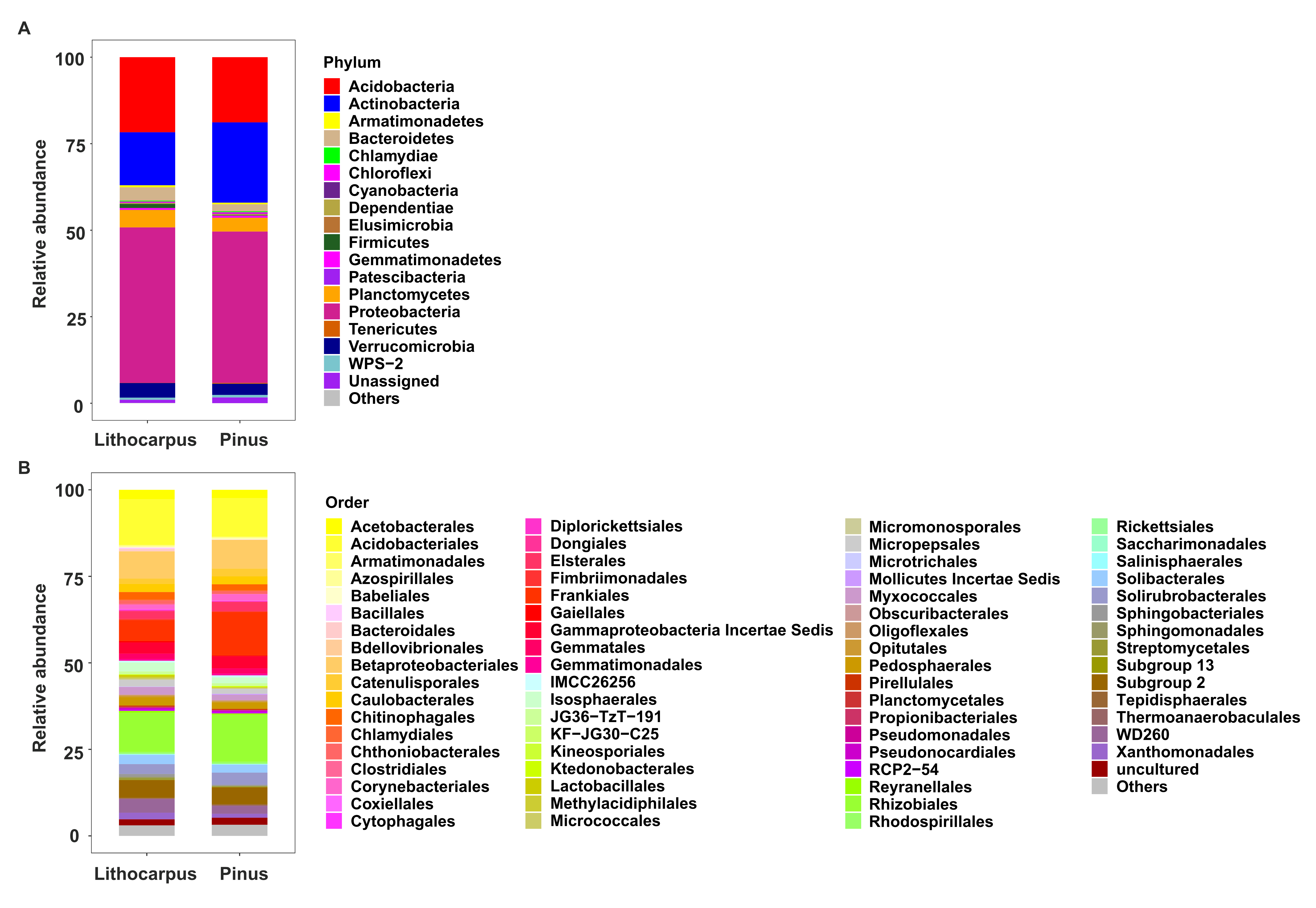

Supplement: SUPPLEMENTARY FIGURE S3 — Relative abundance of the most abundant bacterial OTUs in Lithocarpus and Pinus ECM roots at phylum and order levels. The average relative abundance phyla or class with less than 0.05% across all samples are grouped into “Others”. (A) At phylum level, (B) At order level. [file Image_3.TIF]

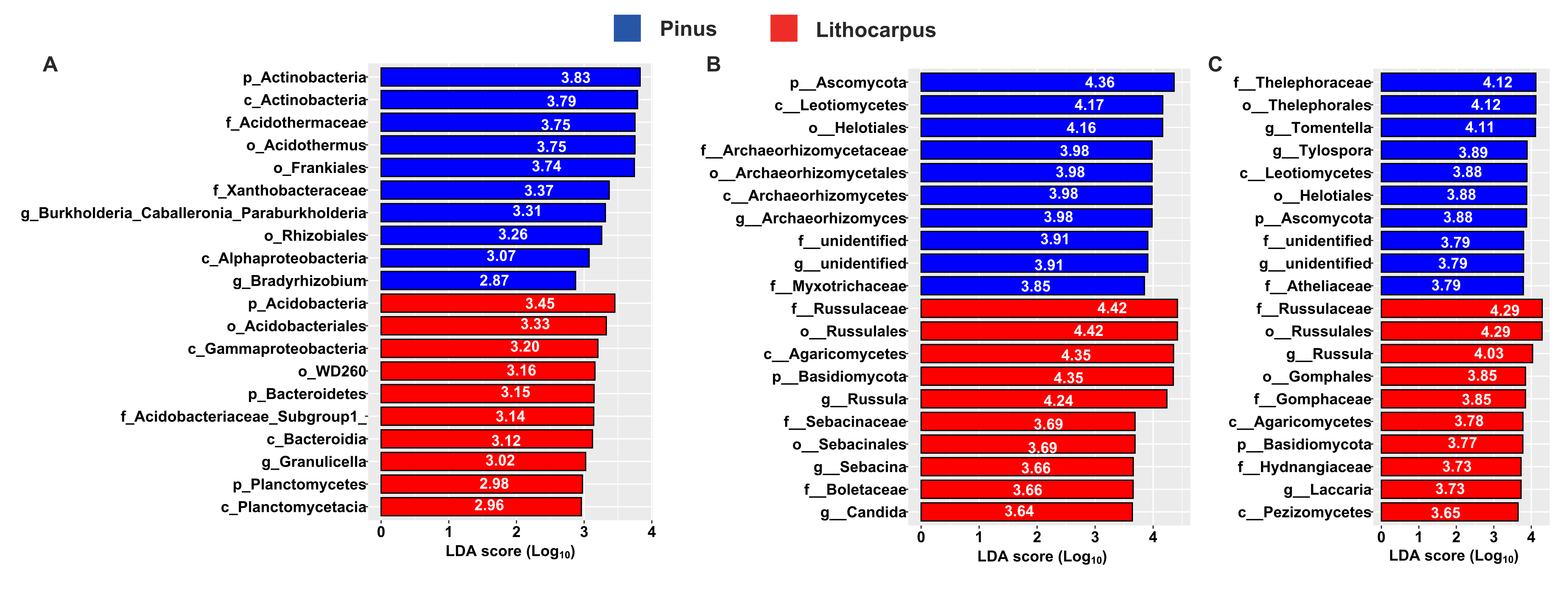

Supplement: SUPPLEMENTARY FIGURE S4 — The biomarker taxa of bacterial and fungal communities varied among Lithocarpus and Pinus ECM roots. LEfSe identified the biomarker taxa associated with Lithocarpus and Pinus ECM roots, respectively. Only the top 10 most specific biomarker taxa are displayed. (A) 16S rDNA sequencing, (B) ITS1 sequencing, (C) ITS2 sequencing. [file Image_4.TIF]

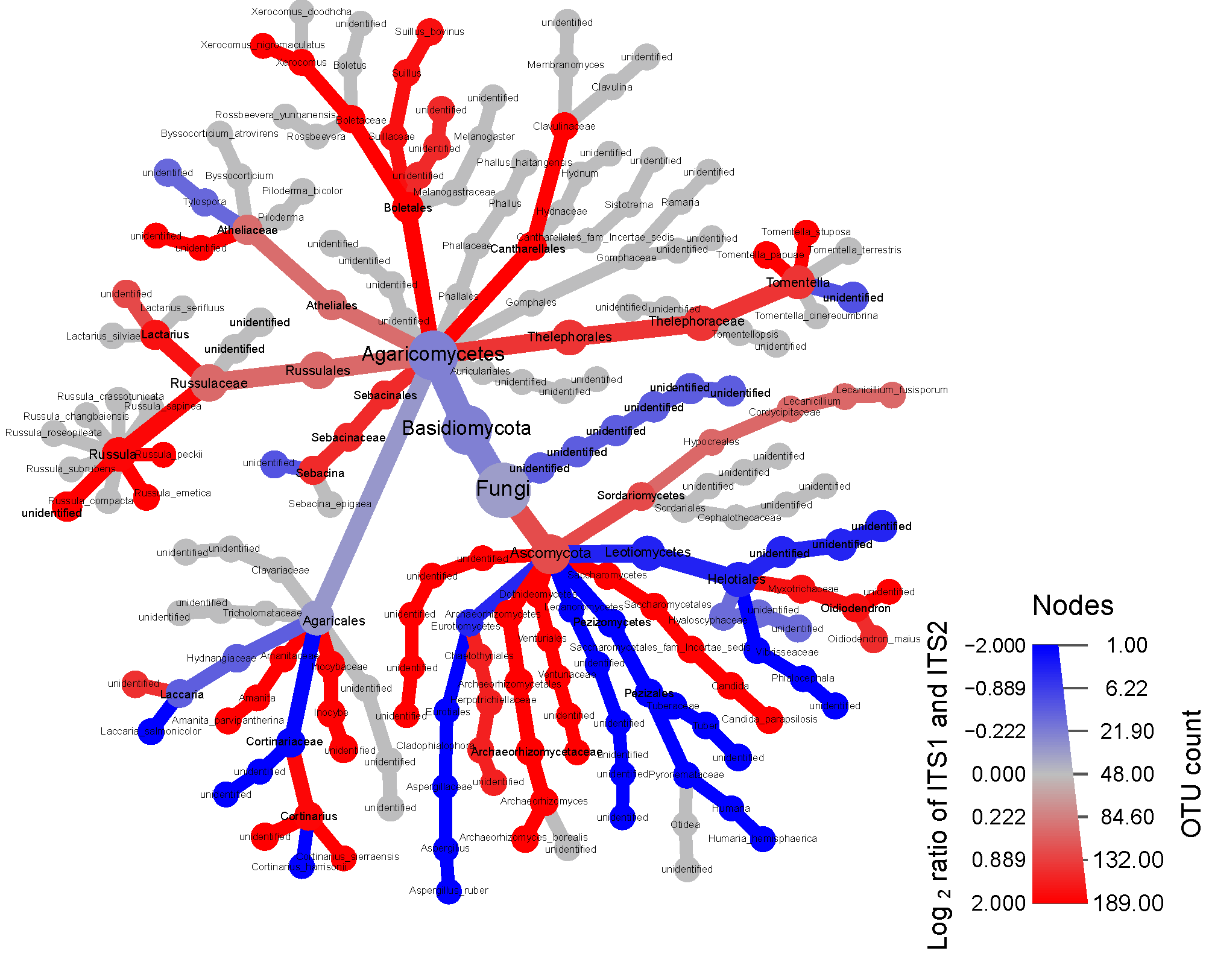

Supplement: SUPPLEMENTARY FIGURE S8 — Taxonomic distribution of the most abundant fungal OTUs identified using either ITS1 or ITS2 sequences. Blue and red circles represent the microbial taxonomic ranks with are found as significantly more abundant based on ITS1 or ITS2 sequences, respectively. The color of nodes represents the value of the Log2 ratio of ITS1 and ITS2 with OTU count (Wilcoxon Rank Sum test, P<0.05). Only the OTUs with an average relative abundance >0.1% are displayed. For these analyses, we used samples collected during the wet seasons. [file Image_8.TIF]

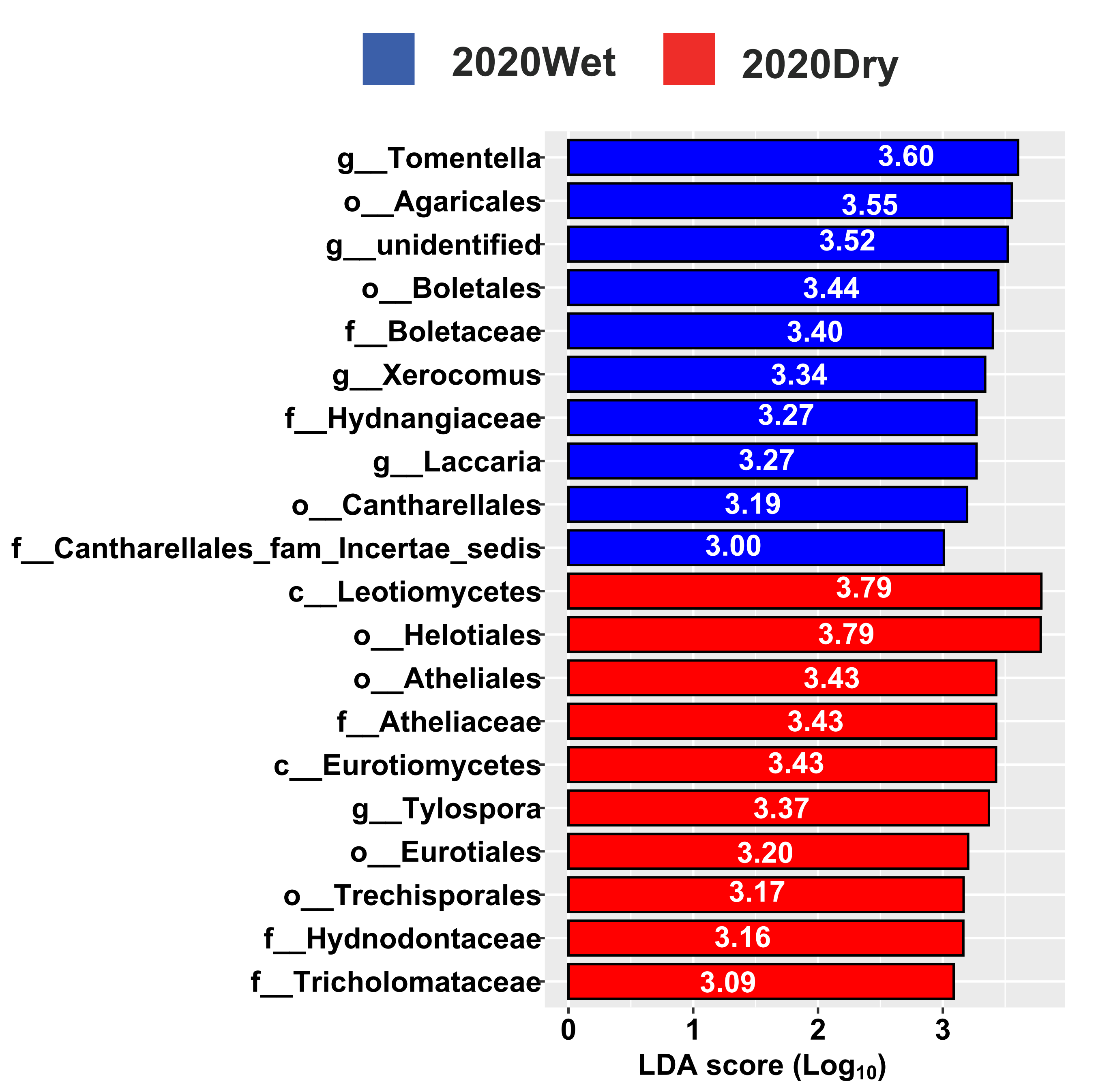

Supplement: SUPPLEMENTARY FIGURE S9 — The biomarker taxa identified in ECM roots at the end of the dry season (2020Dry) and at the end of the wet season (2020Wet) based on ITS1 sequencing. Only the top 10 most specific biomarker taxa are displayed. [file Image_9.TIF]
